# Detection technologies for RNA modifications

**DOI:** 10.1038/s12276-022-00821-0

**Published:** 2022-10-21

**Authors:** Yan Zhang, Liang Lu, Xiaoyu Li

**Affiliations:** 1 Department of Biochemistry and Department of Gastroenterology of the Second Affiliated Hospital, Zhejiang University School of Medicine, Hangzhou, 310058 China; 2 Institute of Immunology, Zhejiang University School of Medicine, Hangzhou, 310058 China

**Keywords:** Biological techniques, Sequencing

## Abstract

To date, more than 170 chemical modifications have been characterized in RNA, providing a new layer of gene expression regulation termed the ‘epitranscriptome’. RNA modification detection methods and tools advance the functional studies of the epitranscriptome. According to the detection throughput and principles, existing RNA modification detection technologies can be categorized into four classes, including quantification methods, locus-specific detection methods, next-generation sequencing-based detection technologies and nanopore direct RNA sequencing-based technologies. In this review, we summarize the current knowledge about these RNA modification detection technologies and discuss the challenges for the existing detection tools, providing information for a comprehensive understanding of the epitranscriptome.

## RNA modifications: Comparing detection tools

Improved methods for detecting chemical modifications of RNA will help to understand the epitranscriptome, the collection of RNA modifications in a cell. Chemical modifications to RNA molecules do not alter the sequence, but can nonetheless greatly affect RNA function. More than 170 RNA modifications have been discovered over the past 60 years and their effects are analogous to epigenetic modifications of DNA. Xiaoyu Li and co-workers at Zhejiang University School of Medicine in Hangzhou, China, reviewed existing tools for detecting RNA modifications. These tools, which are developed based on the inherent chemical properties of RNA modifications, vary in sensitivity, quantification, specificity and accuracy. The researchers also summarize the recent progress in nanopore direct RNA sequencing based detection technology and are optimistic that future advances will enable detection of different modifications simultaneously in the single molecules.

## Introduction

RNA is a single-stranded molecule consisting of four nucleotides: adenosine (A), guanosine (G), cytidine (C) and uridine (U). RNA, both an important and conserved macromolecule, not only participates in the flow of genetic information but also regulates gene expression. Beyond sequence information, chemical modifications add to the complexity of RNA, emerging as a new layer of gene expression regulation. Since the first chemical modification was characterized 60 years ago, more than 170 RNA modifications have been characterized^1^. Most of the RNA modifications have been identified in abundant non-coding RNAs, including ribosomal RNA (rRNA), transfer RNA (tRNA), and small nuclear RNA (snRNA).

The development of detection technologies advances the investigation of the functional roles of RNA modifications^2–7^. To date, more than ten chemical modifications have been mapped in a transcriptome-wide manner, including *N*^6^-methyladenosine (m^6^A), *N*^6^, 2′-O-dimethyladenosine (m^6^Am), 5-methylcytosine (m^5^C), 5-hydroxymethylcytosine (hm^5^C), inosine (I), pseudouridine (Ψ), *N*^1^-methyladenosine (m^1^A), 2ʹ-O-methylation (Nm), *N*^4^-acetylcytidine (ac^4^C), *N*^7^-methylguanosine (m^7^G) and dihydrouridine (D). Different chemical modifications play distinct regulatory roles in RNA metabolism and function. For instance, m^6^A, the most abundant internal messenger RNA (mRNA) modification, influences RNA metabolism in multiple ways, including stability, splicing, translation, localization and RNA secondary structure^8–11^. m^5^C in mRNA influences mRNA export, RNA stability, and translation, and m^5^C in tRNA is essential for maintaining structural stability and translational fidelity^12–19^. Inosine preferentially exists in double-stranded RNA (dsRNA) regions and affects codon recoding, splice-site choice, microRNA (miRNA) biogenesis and targeting efficiency^20–22^. Ψ is required for proper rRNA folding, tRNA structure stabilization and snRNP (small nuclear ribonucleoprotein) biogenesis^23–32^. In addition, introducing Ψ into mRNA increases protein production and alters translation^33–35^. m^1^A at position 58 in tRNA is conserved and vital for stabilizing tRNA tertiary structure, and m^1^A in mRNA influences translation^36–47^. Nm is essential for accurate and efficient protein synthesis^48–52^. Internal m^7^G increases mRNA translation efficiency and augments miRNA biogenesis^53,54^. ac^4^C in mRNA promotes translation, and ac^4^C in rRNA can affect rRNA biogenesis^55–59^.

RNA modification detection technologies provide not only resources for a comprehensive understanding of the epitranscriptome but also tools for functional studies. Hence, in this review, we will summarize the current knowledge about these existing RNA modification detection technologies and discuss the challenges for these existing detection tools. The detection technologies are categorized according to the detection throughput and principles into four classes: quantification methods, locus-specific detection methods, next-generation sequencing-based detection technologies and nanopore direct RNA sequencing-based technologies.

## RNA modification quantification methods

The identification and quantification of new modified nucleotides requires powerful RNA modification quantification methods. Based on the principle that modified nucleotides possess distinct chemical properties from the originals, several RNA modification quantification methods have been established, including two-dimensional thin-layer chromatography (2D-TLC), dot blot, and liquid chromatography–mass spectrometry (LC–MS). These approaches can be used to quantify the modification abundance in specific RNA species and require highly purified RNA due to the lack of sequence information.

### 2D-TLC

**Fig. 1:**
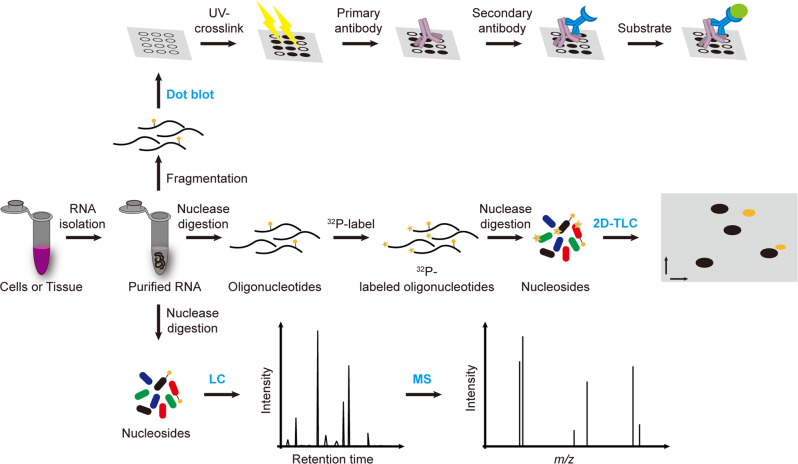
Schemes of RNA modification quantification methods. Technical flowchart of dot blot, 2D-TLC and LC–MS.

2D-TLC is a widely used RNA modification detection method according to the distinct mobilities of different nucleotides in the solvent^60–62^. In detail, isolated RNA is first partially digested into oligonucleotides using RNase A, T1, or T2 and then labeled with ^32^P using T4 polynucleotide kinase (T4 PNK). Finally, 5ʹ-^32^P-NMP is acquired by nuclease P1 digestion and further separated by 2D-TLC. The nucleotides can be determined by assignment to the standards by comparing their retardation factor (Rf) values (Fig. 1). Quantification of the nucleotides is achieved by measuring the radioactivity of the corresponding spots in the TLC plate^63–65^. This approach is very sensitive and requires only a small amount of RNA (from 50 ng to 200 ng). Consequently, this approach can be applied to both abundant non-coding RNAs (rRNA and tRNA) and less abundant mRNA. In addition, it can also detect RNA modifications in specific tRNA or snRNA sequences, which can be isolated by gel purification or hybridization methods. Furthermore, this approach does not require expensive instruments and thus can be run inexpensively. However, this method also has certain drawbacks, including the requirement of a radioactive reagent, the bias caused by RNase digestion and discrepant ^32^P labeling efficiency for the modified nucleotides^63,66^.

### Dot blot

Dot blot assay is established and applied to detect and quantify modification levels in RNAs using a specific antibody. In detail, isolated RNAs are stalled to the polyvinylidene fluoride (PVDF) or nitrocellulose membrane directly without electrophoretic size separation. A specific antibody for target modification is incubated with the membrane, followed by secondary antibody hybridization and subsequent signal trapping (Fig. 1). Comparing results from different experimental groups can obtain semiquantitative modification level information. This approach has been widely applied to various RNA species, including non-coding RNAs and mRNAs. Given the detection principle, the sensitivity and accuracy of this approach are highly dependent on the specificity of the antibody. In addition, the amount of starting material, ranging from nanograms to micrograms, also depends on the abundance of the RNA modifications of interest. Since the workflow is straightforward and can be performed inexpensively, this approach has been widely applied for RNA modification detection^67,68^. However, the lack of absolute quantification and locus information limits a wider application of this approach.

### HPLC and LC–MS

High-performance liquid chromatography (HPLC) is an advanced column chromatography technology to separate nucleosides according to their distinct polarities. Prior to HPLC analysis, RNA or oligonucleotides are digested and dephosphorylated to single nucleosides by nuclease P1 and alkaline phosphatase. The UV absorbance and retention time of the nucleosides are recorded by a UV detector, which can be used to identify and measure the abundance of the modified nucleosides (Fig. 1). Compared with 2D-TLC, HPLC analysis is rapid and free of radiolabeling. However, this approach can only be applied to detect highly abundant modifications in abundant RNA species, such as rRNA, tRNA and synthetic RNAs, and requires a large amount of purified RNA (more than 1 µg) due to the detection limit of the UV detector^69^.

To increase detection sensitivity, HPLC is coupled with mass spectrometry^70^. Similarly, RNA or oligonucleotides are completely digested to nucleosides and separated by reverse column chromatography. Then, these nucleosides are ionized and further fragmented into specific product ions via mass spectrometry. Integration of retention time, mass-to-charge ratio (m/z) and product ion are capable of determining a certain nucleoside (Fig. 1). In addition, quantification of nucleosides can be achieved through the external standard curve in the same batch^60,71^. The extremely high sensitivity of triple quadrupole-based mass spectrometry provides this approach a detection limit that can reach the low femtomolar range, and the amount of starting material can be as low as 50 ng, thereby allowing the determination and quantification of these low-abundance modifications in mRNA and low-abundance ncRNA^72,73^. Hence, LC–MS has been a benchmark for RNA modification detection and quantification. However, the limit of this technique is the requirement of instruments including HPLC and mass spectrometry. In addition, due to the lack of sequence information, when detecting and quantifying RNA modifications in mRNA or other less abundant RNA types, caution must be taken to reduce contamination from abundant and highly modified rRNA and tRNA.

## Locus-specific detection methods

The precise position information of a certain RNA modification is important for functional studies. To date, several locus-specific RNA modification detection methods have been developed. These methods can be categorized according to the detection principles into four classes: (1) Primer extension; (2) RNase H-based approaches; (3) electrospray ionization-mass spectrometry (ESI-MS)-based approaches; and (4) semiquantitative PCR- or qPCR-based approaches.

### Primer extension

**Fig. 2:**
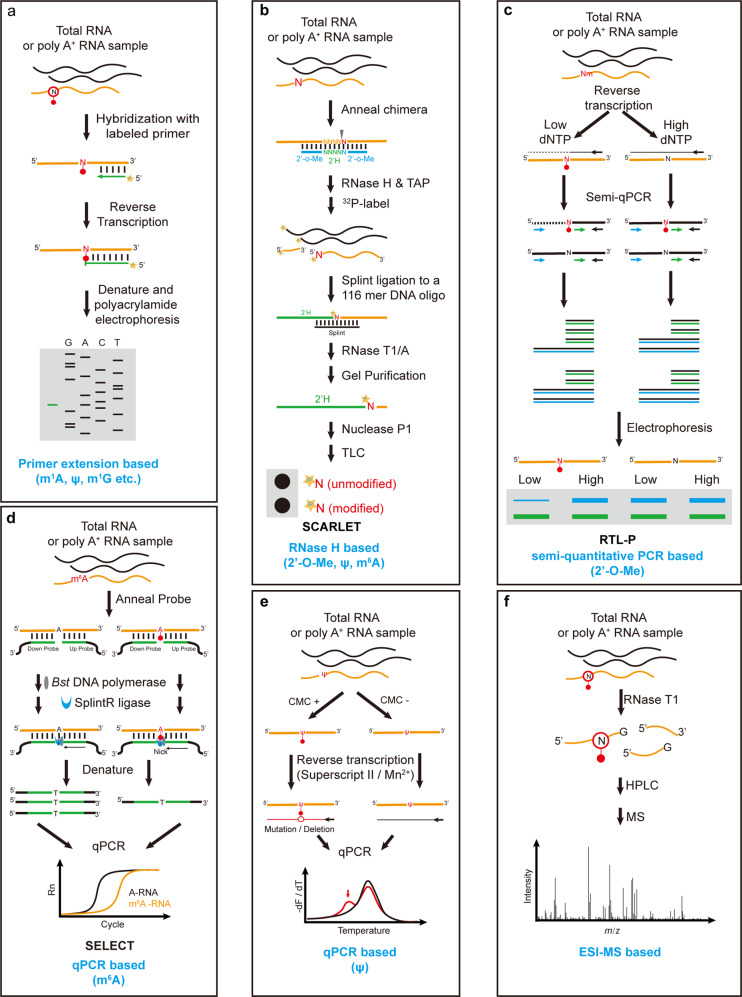
Schemes of locus-specific RNA modification detection methods. **a** Technical flowchart of primer extension. The truncated cDNA product indicates where the modification occurs. **b** Technical flowchart of SCARLET, an RNase H-based approach; the modified and unmodified form of the nucleotide of interest can be accurately quantified by TLC; TAP, thermosensitive alkaline phosphatase. **c** Technical flowchart of RTL-P, a semiquantitative PCR-based approach; Nm impedes RT under low-dNTP conditions and allows readthrough under high-dNTP conditions, and the Nm status can thus be determined by comparing the intensity of the longer PCR product with that of the shorter product. **d** Technical flowchart of the SELECT, qPCR-based approach; this approach utilizes the feature that m^6^A hinders the elongation of *Bst* and reduces the ligation efficiency of SplintR to distinguish modified and unmodified RNA. **e** Technical flowchart of the qPCR-based Ψ detection approach; this approach utilizes the feature that the Ψ-CMC adducts induce mutation/deletion in cDNA, which alters the melting curves of the qPCR products. **f** Technical flowchart of the ESI-MS-based approach. Modifications that can be detected by the corresponding approaches are listed below.

This approach is based on reverse transcription and has been used extensively to detect and localize various RNA modifications, including m^1^A, Ψ, and m^1^G. In principle, a 5ʹ-labeled specific RT primer is hybridized with the RNA of interest and extended by reverse transcriptase. Hence, this approach relies on prior knowledge of the modification type and sequence information of the target RNA. Without modifications, reverse transcriptase can reach the 5ʹ end of RNA and generate full-length cDNA. When encountering modified nucleotides, the extension of reverse transcriptase is blocked immediately upstream of the modified site. Then, the RT products are separated using denaturing polyacrylamide gels, and the terminal position of truncated cDNA indicates where the modification occurred (Fig. 2a). This approach is very sensitive and can be applied to various RNA species, including rRNA, tRNA, snRNA and abundant mRNA^42,43,74–80^. In addition, owing to the hybridization step, this approach possesses high detection specificity and does not require purified homogenous RNA as the starting material. A major limitation of this method is that it can only detect the modifications or their chemical adducts that are capable of blocking reverse transcription; it is not suitable for RT-silent modifications such as m^6^A and m^5^C^81–83^.

### RNase H-based approach

The RNase H-based approach is independent of reverse transcription and thus can detect and quantify RNA modifications that do not affect Watson-Crick base pairing. In this method, purified RNA is cleaved into two halves at the 5ʹ end of the nucleotide of interest by RNase H, and cleavage specificity is achieved by annealing with a specific 2′-O-methyl RNA–DNA chimera oligonucleotide. Then, the 3ʹ-half of the RNA is purified, and its 5′ terminus is further labeled with ^32^P. Furthermore, the oligonucleotides are completely digested into single nucleotides and subsequently resolved by TLC. Owing to the radiolabeling specificity, only the modified and unmodified forms of the nucleotide of interest can be detected and accurately quantified by TLC analysis^64,84^. This approach is sensitive and powerful for quantifying the stoichiometry of modified nucleotides. However, as the specific 2′-O-methyl RNA–DNA chimera oligonucleotide is base-paired with the target RNA sequence to guide RNase H cleavage, sequence information is needed, and modifications that affect Watson-Crick base pairing cannot be detected and quantified by this method. In addition, to reduce false signals from other RNA sequences, homogenously purified RNA, isolated by the hybridization method and gel purification, is used as the starting material. Therefore, this method requires a large amount of RNA and can be applied to abundant non-coding RNAs, such as rRNA and snRNA^64,84^.

To quantitatively detect modifications in mRNA/lncRNA, a new method, named SCARLET (site-specific cleavage and radioactive labeling followed by ligation-assisted extraction and thin-layer chromatography), has been developed^85^. In SCARLET, a 116-nucleotide single-stranded DNA oligonucleotide is splint-ligated to the ^32^P-labeled RNA fragment of interest and then subjected to RNase T1/A digestion. The splint ligation enables RNA mixtures to be used as the starting material rather than a specific purified RNA, and modifications in mRNA and lncRNA can thus also be quantified. The accurate size of the DNA oligonucleotide is recovered by gel purification. Then, the ^32^P-labeled nucleotides are released by nuclease P1 digestion and further analyzed by TLC (Fig. 2b). This approach has been applied to quantify m^6^A and Ψ modification status in mRNA^85,86^. Since this approach avoids reverse transcription, it can be used as an orthogonal method to validate modification sites identified by transcriptome-wide methods that are dependent on reverse transcription. As with other RNase H-based methods, SCARLET also requires prior knowledge of the sequence information to target a single specific nucleotide and cannot quantify the modification status in a de novo manner. In addition, the requirement of radioactive reagents and complicated procedures limit wider application of this method.

### Semiquantitative PCR or qPCR-based approach

Similar to primer extension assays, semiquantitative PCR or qPCR-based approaches are also based on the fact that modified nucleotides impede reverse transcriptase extension. In contrast to primer extension and RNase H-based approaches, qPCR-based approaches are free of radiolabeling and hence are time-saving and easy to perform in the laboratory. To date, several semiquantitative PCR- or qPCR-based approaches have been developed and have successfully detected Nm, Ψ and m^6^A in diverse RNA species (Fig. 2c–e)^87–93^. In addition, owing to the high sensitivity and specificity of PCR and qPCR, these approaches do not require purified RNA as the starting material and can be applied to various RNA species, including less abundant mRNA and lncRNA.

Nm blocks reverse transcriptase extension at a low dNTP concentration and allows readthrough at a high dNTP concentration^82,94^. Taking advantage of this property, researchers have developed a method referred to as RTL-P (reverse transcription at low dNTP concentrations followed by PCR). In this method, the RNA of interest is first reverse-transcribed at both low and high dNTP concentrations. The truncated and full-length cDNA are amplified by specific primers. The PCR products are analyzed by gel electrophoresis, and the Nm status can be determined by comparing the intensity of the longer PCR product with that of the shorter product (Fig. 2c)^87^. In addition to altering RT conditions, RT enzymes can also be engineered to facilitate modified nucleotide detection. For instance, an engineered thermostable KlenTaq DNA polymerase variant possesses reverse transcription activity and can discriminate Nm at normal dNTP concentrations. The combination of this engineered DNA polymerase with qPCR has achieved expeditious quantification of Nm^88^.

In addition to the engineered thermostable KlenTaq DNA polymerase variant, two other DNA polymerases, *Tth* and *Bst*, also have reverse transcriptase activity and exhibit distinct capacities to extend when encountering m^6^A residues versus A residues, which allows locus-specific detection of m^6^A^89–91^. Moreover, to increase the detection sensitivity of m^6^A, another method referred to as the single-base elongation- and ligation-based PCR amplification method (SELECT) has been developed^92,93^. Because m^6^A can both hinder the elongation activity of DNA polymerases and reduce the nick ligation efficiency of SplintR ligase, in SELECT, cDNA products formed from m^6^A-containing RNA templates are dramatically reduced, hence significantly increasing detection robustness. Based on qPCR, SELECT is a powerful tool to quantify the m^6^A fraction in a linear manner (Fig. 2d).

In addition to truncations, the property of induced mutation/deletions by modified nucleotides can also be utilized for locus-specific detection. For instance, Ψ can be selectively labeled by N-cyclohexyl-N′-(2-morpholinoethyl) carbodiimide (CMC), and the Ψ-CMC adducts interfere with Watson-Crick base pairing during reverse transcription. Under optimized RT conditions, Ψ–CMC adducts can be read through and induce mutation/deletion in cDNA. Such mutation/deletions alter the melting curves of the qPCR products, thus enabling the locus-specific detection of Ψ modification (Fig. 2e)^95^.

### ESI-MS-based approach

In contrast to the LC–MS-based nucleoside quantification strategy described above, in this approach, isolated RNA samples are first digested into 5–15 nucleotide fragments by selective endoribonucleases, such as RNase T1, RNase A, and RNase U2. Then, the oligonucleotides are separated by HPLC, and the sequence ladders from the oligonucleotides are further generated through ESI-MS, which can be used for sequence reconstruction and modification identification (Fig. 2f). Hence, this approach can provide both site and stoichiometry information for the modifications of interest^70,96–99^. Compared with other locus-specific methods, the ESI-MS-based approach does not rely on prior knowledge of sequence information and thus is able to detect and quantify RNA modification in a de novo manner. Therefore, this approach has been widely applied for modification detection in abundant RNAs, including tRNA, rRNA and snRNA^100–109^. Recently, this approach has been used to detect modifications in miRNA and cap modifications in mRNA^53,110^. The major limitation of this approach is the requirement for highly sensitive ESI-MS. In addition, given the detection principle, the requirement of starting material is large; thus, this method is only suitable for abundant RNA species.

## Next-generation sequencing-based detection technologies

With the benefit of advances in next-generation sequencing, an increasing number of RNA modification sequencing technologies have been developed. Such technologies represent powerful tools to map modified nucleotides in a transcriptome-wide manner and promote the elucidation of the regulatory roles of RNA modifications. Chemical modifications alter the inherent features of the original nucleotides, including base-pairing performance in reverse transcription, chemical reaction activities, enzymatic reaction activities and binding affinities with specific proteins or antibodies. Therefore, coupled with next-generation sequencing, these properties of modified nucleotides can be used to characterize RNA modifications throughout the transcriptome. The existing sequencing technologies can be categorized according to the detection principles into four classes: (1) direct sequencing technology, (2) chemical-assisted sequencing technology, (3) antibody-based sequencing technology, and (4) enzyme/protein-assisted sequencing technology (Table 1).

**Table 1:** Features of next-generation sequencing-based detection technologies.

Strategy	Method	Modification type	Detection principle	RNA species	Reference
Direct sequencing	RNA and DNA differences (RDD)	Inosine	Comparison of the genomic DNA and RNA sequencing data and detection of A-to-G mismatch sites	total RNA/mRNA/poly(A)- RNA/small RNA (18–30 nt)	^111–114^
	ARM-seq/DM-tRNA-seq	m^1^A, m^3^C, m^1^G, m^2^_2_G, and m^3^U	Detection of mutation signals induced by methylations at the Watson-Crick face and samples treated by WT/mutant AlkB demethylase are used as negative controls to reduce false-positive sites	small RNA (<200 nt)/rRNA/tRNA	^117–120^
	m1A-quant-seq	m^1^A	Evolution of the HIV-1 reverse transcriptase to allow sensitive m^1^A detection in low-abundance RNA and low-stoichiometry sites	mRNA	^122^
	2OMe-seq/MeTH-seq	Nm	Limiting the concentration of dNTP or Mg^2+^ in RT reactions causes RT stops at Nm sites	rRNA/mRNA	^123,124^
	Direct m^6^A Sequencing	m^6^A	Evolution of KlenTaq DNA polymerase as the RTase and induces RT misincorporations at m^6^A sites	model sequence tRNA	^125^
	4SedTTP-involved and FTO-assisted sequencing	m^6^A	Substitution of dTTP with 4SedTTP under RT conditions, which induces RT stops at m^6^A sites; FTO-treated samples are used as negative controls	model sequence	^126^
Chemical-assisted sequencing	BoRed-seq	m^7^G	m^7^G methylation is treated to generate abasic sites and further tagged with biotin molecules, enabling enrichment by streptavidin pulldown	miRNA	^53^
	m6A-SEAL-Seq	m^6^A	m^6^A is first oxidized to hm^6^A by FTO and further converted to dm^6^A through thiol addition; dm^6^A can be conjugated to biotin molecules, thereby enabling streptavidin pulldown	mRNA	^127^
	ICE-seq	Inosine	Inosine can be selectively labeled with acrylonitrile, and the resulting ce^1^I induces RT stops	mRNA	^128^
	RNA-BisSeq/BS-seq	m^5^C	Compared with unmodified C, m^5^C is resistant to bisulfite treatment and thus can be characterized by detecting nonconverted Cs	mRNA/total RNA/large RNA (>200 nt)/small RNA (<200 nt)	^16,129–133^
	Ψ-Seq/Pseudo-seq/PSI-seq	Ψ	Ψ can be labeled by CMC, and the resulting CMC-Ψ adducts induce RT stops	mRNA/total RNA/tRNA	^134–136^
	CeU-seq	Ψ	Ψ is labeled with N_3_-CMC and further conjugated with biotin, thereby enabling preenrichment of Ψ-containing RNA and further base-resolution detection of low-stoichiometry Ψ sites	mRNA	^86^
	RBS-seq	m^5^C, m^1^A, Ψ	Optimization of bisulfite treatment conditions and simultaneous detection of all three modifications in the same RNA	mRNA/rRNA/ tRNA	^131^
	m^7^G-MaP-seq	m^7^G	m^7^G residues can be converted to abasic sites upon NaBH_4_ treatment and further recorded as misincorporations through reverse transcription	rRNA/tRNA	^137^
	m^7^G-seq	m^7^G	m^7^G residues are reduced to abasic sites and further generate biotinylated AP sites, which can be enriched by biotin pulldown and induce RT stops when using HIV reverse transcriptase	mRNA/rRNA/tRNA	^54^
	Rho-seq	D	D can be reduced by NaBH_4_ treatment and further labeled by Rho, which can induce RT stops in cDNA synthesis	mRNA/tRNA	^138^
	ac4C-seq	ac^4^C	ac^4^C can be reacted with NaCNBH_3_ under acidic conditions to form N4-acetyltetrahydrocytidine, which induces misincorporation during cDNA synthesis	total RNA/mRNA	^139^
	m^6^A-label-seq	m^6^A	m^6^A is converted to a^6^A by metabolic labeling with Se-allyl-L-selenohomocysteine, and a^6^A RNA can be further converted to cyc-A, which can induce misincorporations during cDNA synthesis	mRNA	^140^
	m6A-SAC-seq	m^6^A	m^6^A is converted to a^6^m^6^A by specific dimethyltransferases, and cyclized a^6^m^6^A can induce misincorporation during cDNA synthesis	mRNA/rRNA-depleted RNA	^141^
	RiboMeth-seq	Nm	Nm is resistant to alkaline hydrolysis; therefore, it can be mapped by analyzing read-end information in sequencing data	rRNA	^142–144^
	RibOxi-seq/Nm-seq	Nm	Treatment with iterative OED cycles to remove unmodified nucleotides and selective ligation of Nm-modified ends	mRNA/rRNA	^145,146^
	AlkAniline-Seq/TRAC-seq	m^3^C, m^7^G	m^3^C and m^7^G is resistant to NaBH_4_-aniline treatment and cleavage, thereby enabling selective ligation to enrich modified fragments	rRNA/tRNA	^147,148^
	HAC-seq	m^3^C	m^3^C-modified sites can be specifically cleaved upon hydrazine/aniline treatment and can therefore be mapped by calculating the cleavage ratio	tRNA/ rRNA depleted total RNA	^149^
	HydraPsiSeq	Ψ	Ψ modified sites are resistant to hydrazine/aniline-induced cleavage, and the protection signal can be identified by comparing the cleavage efficiency of neighboring unmodified U sites	rRNA/mRNA	^150^
Antibody-based sequencing	m^6^A-seq/ MeRIP	m^6^A/m^6^Am	Certain modification-containing RNA fragments can be enriched by specific antibody immunoprecipitation	total RNA/mRNA	^151,152^
	m^1^A-ID-seq/ m^1^A- seq	m^1^A		mRNA	^39,153^
	hMeRIP	hm^5^C		mRNA	^154^
	acRIP-seq	ac^4^C		mRNA	^55^
	m7G-RIP-Seq/m7G-Seq/ m^7^G MeRIP	m^7^G		mRNA/ small RNA (<200 nt)/ miRNA	^53,54,148^
	miCLIP-seq/ PA-m6A-seq	m^6^A	Introducing UV-induced RNA-antibody crosslinking around modified sites, thereby inducing truncations or misincorporations during cDNA synthesis	mRNA	^155,156^
	m^7^G miCLIP-seq	m^7^G		mRNA	^157^
	m^1^A-MAP/m^1^A-seq/m^1^A-IP-Seq	m^1^A	Coupling m^1^A immunoprecipitation with enzyme/chemical treatment and taking advantage of m1A-induced mutational RT signatures to achieve single-base resolution detection	mRNA	^42,122,158^
	m^6^Am-seq	m^6^Am	Selective in vitro demethylation for m6Am to discriminate m^6^Am from m^6^A	>200 nt RNA	^159^
Enzyme/protein-assisted sequencing	AZA-IP	m^5^C	Metabolic labeling with 5-aza-C to form a covalent bond with m^5^C methyltransferase, thereby enabling immunoprecipitation of the direct targets of m^5^C methyltransferases	total RNA	^161^
	m^5^C-miCLIP	m^5^C	Overexpression of mutated Nsun2, which can form covalent bonds with its target sites	total RNA	^160^
	DART-seq	m^6^A	Fusion of APOBEC1 to the m^6^A-binding YTH domain, thereby leading to C-to-U mutation adjacent to m^6^A residues	total RNA	^162^
	scDART-seq	m^6^A	Integration of DART-seq with a single-cell RNA-sequencing platform to achieve profiling of the m^6^A methylome in single cells	total RNA	^163^
	EndoVIPER-seq	Inosine	In the presence of Ca^2+^, *E. coli* Endonuclease V prefers binding to inosine in RNA, which enables high-affinity capture of inosine	mRNA	^164^
	m^6^A–REF-seq/MAZTER-seq	m^6^A	MazF can specifically cleave the unmethylated 5ʹ-ACA-3ʹ motif to distinguish m^6^A from A	mRNA	^166,167^

### Direct sequencing technology

For some modified nucleotides, the existence of chemical modifications alters canonical Watson-Crick base pairing during reverse transcription and further leads to truncation or misincorporation in cDNA synthesis. Therefore, this feature can be used to map modified nucleotides throughout the transcriptome.

**Fig. 3:**
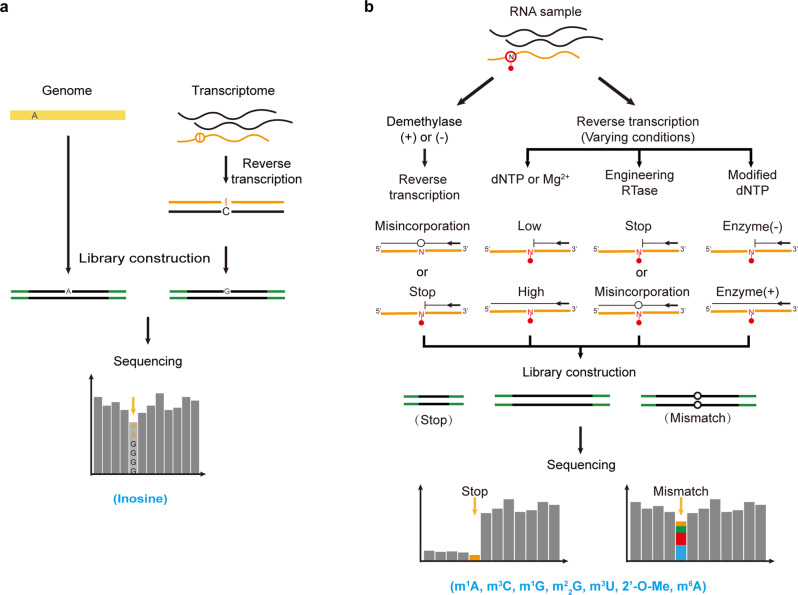
Schemes of direct sequencing technologies. **a** Detection of A-to-I editing sites by detecting A-to-G mismatches between genomic DNA and RNA sequencing (RNA-seq) data from the same individuals. **b** Detection of modified nucleotides by utilizing demethylase treatment, altered RT conditions, engineered reverse transcriptase, or modified dNTPs to generate distinct RT signatures at modified sites between treated and untreated samples. Modifications that can be detected by the corresponding approaches are listed below.

For instance, in contrast to adenosine, inosine pairs with cytidine in reverse transcription, and thus, the A-to-I editing position can be identified by comparing the genomic DNA and RNA sequencing data and detecting A-to-G mismatch sites (Fig. 3a)^111–114^. Although this approach is widely applied to A-to-I editing site detection, caution is still needed to reduce the false positives introduced by SNPs, somatic mutations, pseudogenes and sequencing errors^22^.

Aside from inosine, several other chemical modifications at the Watson-Crick face of the nucleobase can also induce RT stops or mutations in cDNA synthesis, thereby allowing base-resolution detection^115^. In contrast to inosine, the reverse transcription signatures of these modifications are not constant and dependent on both the surrounding sequences and the RT conditions^116^. Furthermore, to increase the detection accuracy, several improvements have been made. For instance, the *E. coli* AlkB demethylase and its mutant treatment are introduced to remove m^1^A, m^3^C, m^1^G, *N*^2^,*N*^2^-dimethyl-guanosine (m^2^_2_G), and 3-methyluridine (m^3^U) in RNA prior to cDNA synthesis, and high-confidence methylation sites in the tRNA transcriptome can thus be identified by comparing the data for parallel sequenced, demethylase-treated and untreated samples (Fig. 3b)^117–120^. Moreover, the RT signatures vary substantially under different reverse transcription conditions^42,121^. Hence, to improve detection sensitivity, an HIV-1 reverse transcriptase against m^1^A was developed that allows m^1^A detection in human mRNA^122^. Moreover, RT systems, including enzymes and reaction conditions, can be optimized to detect modified nucleotides that do not interfere with Watson-Crick base pairing. For instance, Nm, an RT-silent modification, can block cDNA synthesis when the concentration of dNTPs or Mg^2+^ in RT reactions is limited. Coupling this feature with next-generation sequencing, two methods, 2OMe-seq and MeTH-seq, have achieved a transcriptomic profile of Nm at base resolution (Fig. 3b)^123,124^. As mentioned above, some DNA polymerases also possess reverse transcriptase activity and show differential elongation ability when encountering m^6^A residues versus A residues. To advance this feature, the KlenTaq DNA polymerase was evolved and the evolved variant exhibited significantly increased error rates opposite m^6^A but not unmodified A, enabling direct identification of m^6^A by analyzing the mutational signal from sequencing data (Fig. 3b)^125^. In addition, substitution of 4SedTTP (atom-specific replacement of oxygen with selenium at the 4-position) for dTTP under RT conditions can also facilitate m^6^A detection. In principle, compared with 4SeT-A, 4SeT-m^6^A pairing is unfavorable and results in aborted cDNA synthesis opposite m^6^A sites, thereby allowing for the discrimination of m^6^A from A. Furthermore, with the assistance of the m^6^A demethylase FTO through high-throughput sequencing, m^6^A can be precisely identified within the mammalian transcriptome at single-nucleotide resolution (Fig. 3b)^126^.

### Chemical-assisted sequencing technologies

**Fig. 4:**
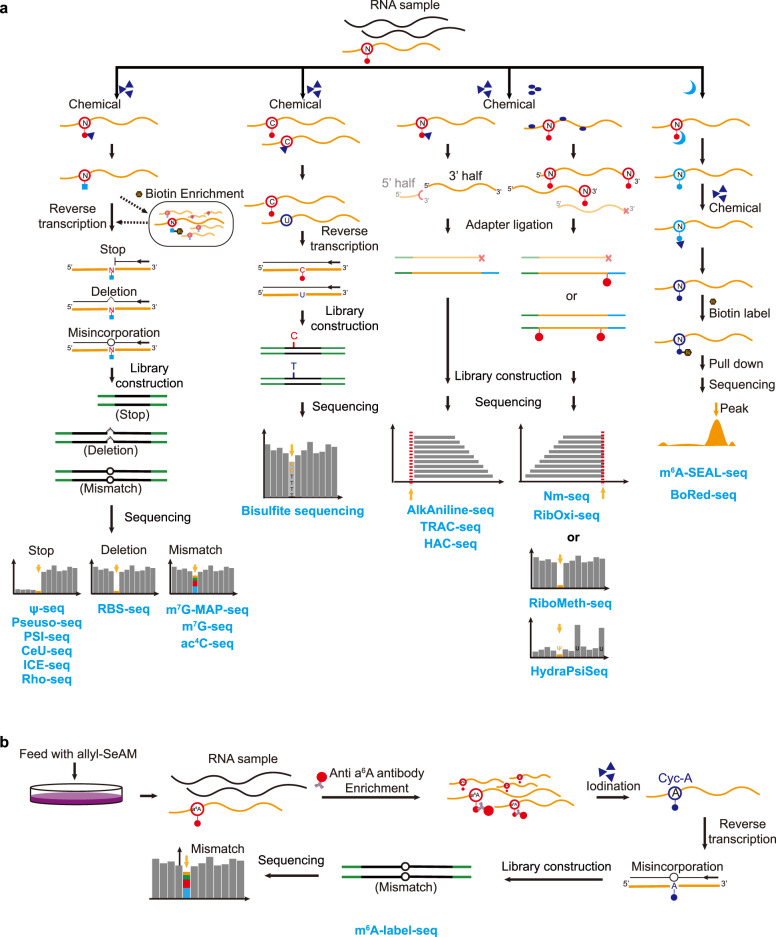
Schemes of chemical-assisted sequencing technologies. **a** Schematic diagrams and sequencing features of different technologies. Corresponding technologies are listed below. **b** Flowchart of m6A-label-seq.

Chemical treatments are widely exploited to discriminate modified nucleotides from unmodified nucleotides in three ways: (1) installing biotin tags to enrich modified transcripts; (2) altering the base-pairing features to induce misincorporation or truncation in reverse transcription; and (3) chemical-induced cleavage followed by specific adaptor ligation (Fig. 4).

The enrichment strategy facilities RNA modification detection in low-abundance RNA species and low-stoichiometry modifications. Some modified nucleotides can be labeled by specific chemical reactions and further conjugated with the biotin molecules, thus enabling streptavidin enrichment. For instance, in borohydride reduction sequencing (BoRed-seq), total RNA is treated with NaBH_4_ and subsequently exposed to low pH to generate abasic sites at m^7^G positions, which can be tagged with biotin molecules for further streptavidin pulldown (Fig. 4a)^53^. Direct m^6^A labeling is difficult due to the inert reactivity of the methyl group. To solve this challenge, researchers developed a method named m^6^A-SEAL-Seq (an FTO-assisted m^6^A selective chemical labeling method). In this method, m^6^A is first oxidized to hm^6^A by the demethylase FTO, and hm^6^A is further converted to *N*^6^-dithiolsitolmethyladenosine (dm^6^A) through DTT-mediated thiol addition. The free sulfhydryl group of dm^6^A enables biotin to be conjugated to the m^6^A-modified transcripts, thereby facilitating streptavidin enrichment and sequencing (Fig. 4a)^127^.

The detection strategy of altering the base-pairing properties of nucleotides by chemical treatment has been widely applied to the transcriptome-wide detection of various modifications, including inosine, m^5^C, Ψ, m^7^G, ac^4^C, D and m^6^A. For inosine detection, the direct sequencing approach is disturbed by background noise. To overcome this limitation, a selective chemical labeling reaction with acrylonitrile is adopted, and the formed *N*^1^-cyanoethylinosine (ce^1^I) blocks reverse transcription, resulting in truncation of cDNA. Coupling this chemical reaction with sequencing, referred to as inosine chemical erasing sequencing (ICE-seq), can achieve base-resolution inosine detection throughout the transcriptome (Fig. 4a)^128^. Bisulfite treatment selectively converts unmethylated cytidine into uridine, leading to a C-to-T transition in the sequencing data; thus, transcriptome-wide m^5^C can be characterized by detecting nonconverted Cs in the sequencing data (Fig. 4a)^129,130^. Furthermore, to reduce false positives caused by insufficient conversion, several improvements have been exploited, including optimizing bisulfite treatment conditions to increase deamination efficiency^131,132^, using ACT random hexamers devoid of Gs to avoid copying inefficiently deaminated RNA templates^16^, and developing robust computational pipelines to accurately identify m^5^C sites^132,133^. Ψ can be labeled by CMC at the Watson-Crick face, and the CMC-Ψ adducts stall reverse transcription, thus inducing truncations in cDNA synthesis. Combining this chemical reaction with next-generation sequencing, researchers developed Ψ-Seq, Pseudo-seq and PSI-seq, achieving base-resolution pseudouridylation detection in yeast and mammalian transcriptomes^134–136^. To improve the robustness of Ψ detection, the chemical reaction is adapted by using a synthesized CMC derivative, azido-CMC (N_3_-CMC), instead of CMC. The presence of an azido group enables conjugation with biotin through a click reaction. Hence, the Ψ-containing RNA can be pre-enriched before sequencing, and this method is named CeU-seq^86^. The pre-enrichment step enables CeU-seq to identify thousands of Ψ sites in the mammalian transcriptome (Fig. 4a). In addition to CMC labeling, a recent work showed that Ψ can form a stable monobisulfite adduct upon bisulfite treatment and further leave a deletion signature at the exact modified sites, thereby providing an orthogonal Ψ detection strategy (Fig. 4a)^131^. As described above, m^7^G residues can be converted to abasic sites upon NaBH_4_ treatment and further recorded as misincorporations through reverse transcription and sequencing. Accordingly, the m^7^G Mutational Profiling sequencing (m^7^G-MaP-seq) can map internal m^7^G modifications at nucleotide resolution in tRNA and rRNA^137^. To achieve m^7^G detection in mRNA, m^7^G-seq conjugates a biotin molecule to the generated abasic sites, thus enabling pre-enrichment of m^7^G-containing RNA. Moreover, the biotinylated sites induce misincorporations during cDNA synthesis; therefore, transcriptome-wide base-resolution m^7^G mapping can be achieved (Fig. 4a)^54^. In addition, D can also be reduced by NaBH_4_ treatment, and the reduction product can be further labeled by Rho (rhodamine). Furthermore, the Rho-adducts block reverse transcriptase elongation and thus can be identified by analyzing induced RT-stops in sequencing data (Fig. 4a)^138^. ac^4^C can react with NaCNBH_3_ under acidic conditions, and the formed reduced nucleobase, *N*^4^-acetyltetrahydrocytidine, causes misincorporation during cDNA synthesis. Taking advantage of this reaction, ac^4^C-seq can map ac^4^C at single-nucleotide resolution (Fig. 4a)^139^. In addition to altering the structure of nucleotides by chemical reactions, the chemical group can also be introduced through metabolic labeling. For instance, when feeding cells with an S-adenosyl methionine (SAM) analog, Se-allyl-L-selenohomocysteine, the cellular RNAs could be modified with *N*^6^-allyladenosine (a^6^A) at supposed m^6^A-generating sites. Furthermore, a^6^A-containing RNAs can be enriched by a specific antibody, and a^6^A sites are converted to *N*^1^,*N*^6^-cyclized adenosine (cyc-A) through the iodination-induced cyclization reaction. As cyc-A induces misincorporations in cDNA synthesis, m^6^A can be mapped at base resolution by detecting mutation signals in sequencing data (Fig. 4b)^140^. With the exception of metabolic labeling, the allyl group can also be transferred to m^6^A by the Dim1/KsgA family of dimethyltransferases, which can specifically convert m^6^A into allyl-modified m^6^A (*N*^6^-allyl, *N*^6^-methyladenosine, a^6^m^6^A). Since cyclized a^6^m^6^A can induce misincorporation in RT, the technology, named m^6^A-SAC-seq, achieves quantitative, transcriptome-wide mapping of m^6^A at single nucleotide resolution^141^.

Compared with unmodified nucleotides, modified nucleotides exhibit distinct resistance under chemical hydrolysis treatment. Based on this principle, several sequencing methods have been developed and have achieved transcriptome-wide profiling for Nm, m^7^G, m^3^C and Ψ. Nm is resistant to alkaline hydrolysis and thus can be mapped at single-base resolution by analyzing read-ends information in sequencing data. Since RNA fragmentation is random and irregular, this strategy requires rather high read coverage and is limited to highly abundant RNAs^142–144^. To overcome this limitation, ribose oxidation sequencing (RibOxi-seq) and Nm-seq have been developed. In these two methods, RNA is treated with iterative oxidation–elimination–dephosphorylation (OED) cycles to remove unmodified nucleotides, and the non-methylated ends cannot be ligated to linkers for sequencing library construction^145,146^. The selective cleavage and ligation allow detection of low-stoichiometry 2ʹ-O-methylation sites using this approach. Similarly, m^7^G and m^3^C are resistant to NaBH_4_-aniline treatment, and the generated 5′-phosphate end during aniline cleavage could be exploited for selective ligation to enrich modified fragments^147,148^. This positive selection strategy facilitates transcriptome-wide detection of m^7^G and m^3^C. In addition, upon hydrazine-aniline treatment, RNA can be specifically cleaved at m^3^C-modified sites and resistant to cleavage at Ψ-modified sites. Based on this approach, hydrazine-aniline cleavage sequencing (HAC-seq) and HydraPsiSeq have been developed to detect m^3^C and Ψ, respectively (Fig. 4a)^149,150^.

### Antibody-based sequencing technologies

**Fig. 5:**
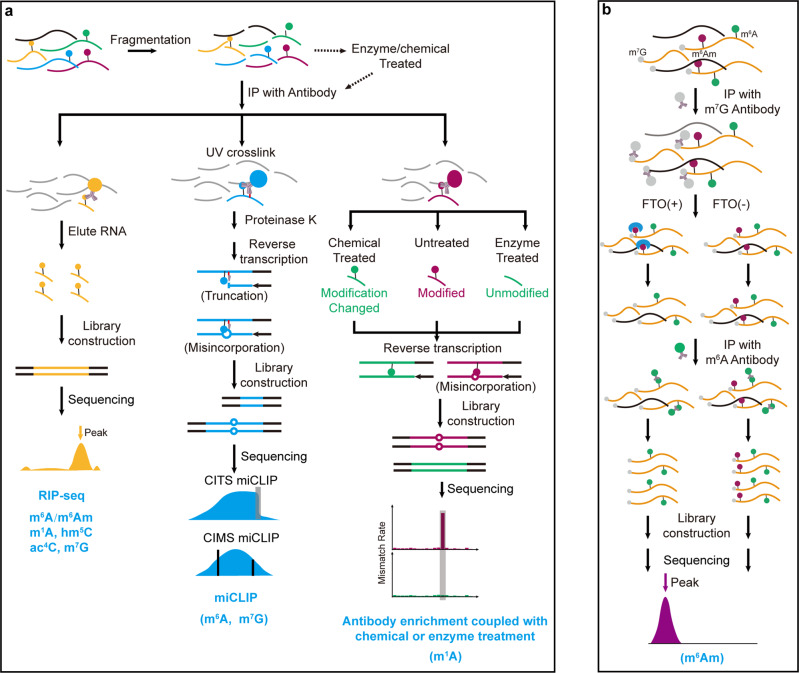
Schemes of antibody-based sequencing technologies. **a** Schematic diagrams and sequencing features of RIP-seq, miCLIP and antibody-based technologies coupled with chemical/enzyme treatment technologies. Modifications that can be detected by the corresponding approaches are listed below. **b** Flowchart of m^6^Am-seq.

Antibody-based strategies have been exploited for transcriptome-wide mapping of several RNA modifications, including m^6^A/m^6^Am, m^1^A, hm^5^C, ac^4^C and m^7^G (Fig. 5)^39,53–55,148,151–154^. In this strategy, isolated RNA is first fragmented to 100–200 nt, and certain modification-containing RNA fragments are enriched by specific antibody immunoprecipitation. The enriched RNAs are subjected to high-throughput sequencing, and the modifications of interest can be identified by bioinformatic analysis. Robust enrichment allows antibody-based strategies to be very sensitive in detecting low-abundance modifications in mRNA and other rare RNA species.

However, limited by the fragmentation size, the resolution of the antibody-based strategy is approximately 100–200 bp. To improve the detection resolution, several improvements have been made. For instance, introducing UV-induced RNA-antibody crosslinking can lead to truncation or misincorporation at protein–RNA crosslinking sites during reverse transcription, thereby allowing transcriptome-wide single-base resolution detection for m^6^A and m^7^G, respectively (Fig. 5a)^155–157^. In addition, coupling antibody immunoprecipitation with enzyme/chemical treatment can not only increase detection resolution but also reduce false positives. For example, taking advantage of the fact that the m^1^A-induced mutational RT signatures can be erased by AlkB demethylase treatment or Dimroth rearrangement, m^1^A-MAP, m^1^A-seq and m^1^A-IP-Seq have achieved base-resolution m^1^A methylome detection (Fig. 5a)^42,122,158^. In addition, utilizing selective in vitro demethylation for m^6^Am, m^6^Am-seq has the capability of discriminating m^6^Am from m^6^A and can identify m^6^Am at base resolution (Fig. 5b)^159^.

## Enzyme/protein-assisted sequencing technologies

**Fig. 6:**
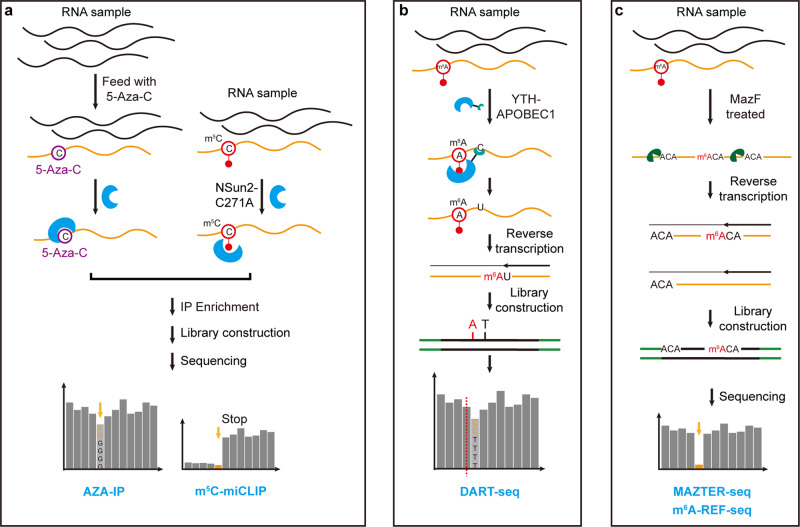
Schemes of enzyme/protein-assisted sequencing technologies. Flowchart of **a** AZA-IP and m5C-miCLIP; **b** DART-seq; **c** MAZTER-seq and m^6^A-REF-seq.

In addition to antibody immunoprecipitation, some enzymes or RNA modification-related proteins can also be utilized for affinity capture or editing modification-containing transcripts, thereby enabling transcriptomic RNA modification detection (Fig. 6). For instance, by feeding a 5-aza-C analog or overexpressing mutated Nsun2, a covalent bond can be formed between m^5^C methyltransferase and its target sites. Based on this approach, Aza-IP (5-azacytidine–mediated RNA immunoprecipitation) and miCLIP (methylation iCLIP) can enrich target sites by immunoprecipitation, thereby enabling identification of the direct targets of m^5^C methyltransferases (Fig. 6a)^160,161^. In addition to methyltransferase, reader proteins can also be used to target modified nucleotides. For example, in DART-seq (deamination adjacent to RNA modification targets), the cytidine deaminase APOBEC1 is fused to the m^6^A-binding YTH domain and thus leads to C-to-U deamination at sites adjacent to m^6^A residues. Furthermore, m^6^A residues can be identified by analyzing C-to-T mismatches in sequencing data (Fig. 6b)^162^. Recently, the authors further integrated DART-seq with a single-cell RNA-sequencing platform and thus developed scDART-seq, achieving profiling of the m^6^A methylome in single cells^163^. In addition, some RNA exonuclease also possesses binding affinity for certain RNA modifications under certain conditions. For instance, in the presence of Ca^2+^, *E. coli* Endonuclease V (eEndoV) promotes binding of inosine in RNA instead of cleavage. Taking advantage of this approach, Endonuclease V immunoprecipitation enrichment sequencing (EndoVIPER-seq) can enrich A-to-I edited transcripts from cellular RNA^164^.

Similar to chemical-induced hydrolysis, some endonucleases also show distinct cleavage efficiency between modified and unmodified transcripts, which can be utilized to enrich modification-containing transcripts and identify modified nucleotides. For instance, the *E. coli* RNA endoribonuclease MazF can specifically cleave the unmethylated 5ʹ-ACA-3ʹ motif but not the 5ʹ-m^6^ACA-3ʹ motif^165^. Taking advantage of this specificity, RNA-endoribonuclease–facilitated sequencing (m^6^A–REF-seq)/MAZTER-seq allows quantitative profiling of m^6^A at single-nucleotide resolution (Fig. 6c)^166,167^. However, a major limitation of this approach is that it can only detect m^6^A in the ACA context, which is only a small portion (16-25%) of m^6^A-modified sites.

## Nanopore direct RNA sequencing-based detection technology

**Fig. 7:**
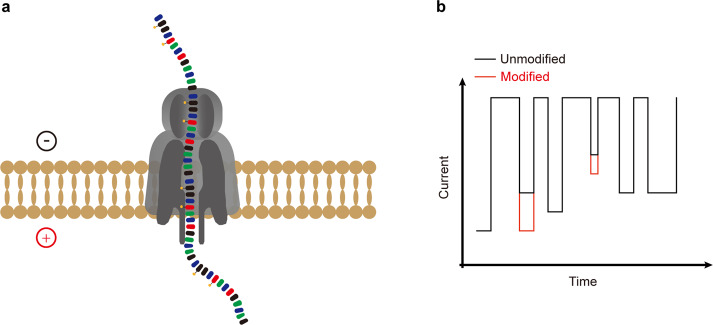
Schemes of nanopore direct sequencing-based detection technology. **a** Ionic current changes caused by a single RNA molecule passing through the nanopore. **b** Current fluctuation caused by modified nucleotides is distinct from that of unmodified nucleotides.

Next-generation sequencing-based detection technologies have been widely applied for transcriptome-wide RNA modification detection. However, limited by the sequencing length (from 50 to 300 bp) and distinct detection principles for different modifications, next-generation sequencing-based detection technologies cannot map diverse RNA modifications simultaneously. The development of the Oxford Nanopore Technologies (ONT) sequencing platform shows promise in overcoming these challenges. In contrast to next-generation RNA sequencing, nanopore sequencing can sequence RNA directly without the requirement of additional reverse transcription and PCR amplification, thus decreasing the biases caused by these steps^168–171^. Mechanistically, single-stranded RNA is driven through the nanopore by the motor protein and thus causes ionic current changes for a set of k nucleotides residing within the pore (kmer; typically, k is 5), which enables decoding of the nucleotide sequence by computational analysis (Fig. 7). In addition to sequence information, chemical modifications and secondary structure, which also influence RNA translocation in nanopores, can be determined directly by computational algorithms^172^. In addition, the reads generated by nanopore sequencing are long enough to cover the full length of a transcript, thereby enabling accurate identification of highly repetitive regions, spliced products and polyadenylation tail length.

To identify RNA modification from the nanopore RNA direction, robust computational algorithm analysis is vital. There are two major analysis strategies, including alterations of raw signal intensity analysis (signal intensity, dwell time and trace) and base-called “error” feature analysis (base quality, mismatch frequency, and deletion frequency)^170^. Furthermore, modifications can be identified by the algorithms previously trained with modified and unmodified kmer contexts or comparison with the nonmodified control samples. These computational algorithms have allowed the identification of several modifications, including m^6^A, m^7^G, m^5^C, hm^5^C, Ψ and Nm, using nanopore direct RNA sequencing^169,172–180^. In addition, owing to the long sequencing length, the sequence information and modification landscape of SARS-CoV-2 can be determined simultaneously by nanopore direct RNA sequencing^181,182^. However, there are still many challenges and limitations for nanopore RNA sequencing-based detection technology. First, the sequencing error rate (~1–4%)^168^ is still much higher than that of next-generation sequencing (~0.1-1%). Since some algorithms exploit systematic base-calling errors to identify RNA modifications, the high sequencing error rate increases the complexity for base calling and modification identification. Second, the throughput of nanopore RNA sequencing is relatively low (1–3 Gb per flow cell), and the costs of direct RNA sequencing are high. Recently, several studies have shown that accurate modification detection requires high sequencing coverage (at least 30X)^173,180^. Hence, the sequencing depth and costs limit broader application of this strategy. Third, nanopore direct RNA sequencing requires a large amount of RNA (~500 ng polyA+ RNA), and thus, it is not suitable for samples that are difficult to obtain. Therefore, efforts are still needed to improve nanopore RNA direct sequencing, including the development of more powerful nanopore proteins and robust computational algorithms.

## Discussion

Since all existing detection methods confer both advantages and drawbacks, appropriate detection strategies should be chosen by considering the abundance of RNA modifications, RNA types of interest (rRNA, tRNA, mRNA or miRNA), the amount of starting material, etc. For instance, direct sequencing methods preserve the modification status but are often confounded by other factors, including RNA secondary structure, SNPs, and sequencing errors. Direct sequencing methods also require a high sequencing depth to detect modifications in low-abundance RNA. Antibody-based detection technologies are powerful and capable of detecting low-abundance modifications in rare RNA species. However, due to the enrichment step, the stoichiometry information of modification is lost, and large starting materials are needed. In addition, since antibodies may have intrinsic bias on RNA sequences and secondary structures, caution is needed to eliminate false-positive signals. Chemical treatment-based approaches have been widely applied to RNA modification detection owing to their high labeling specificity. For this strategy, both high labeling efficiency and mild reaction conditions are required for transcriptome-wide modification detection and quantification. Metabolic labeling or RNA modification-related protein-based approaches provide an alternative strategy to characterize modified residues but can only be applied to cultured cells. Therefore, appropriate negative controls are necessary to reduce false positives, including modification status manipulation (enzyme knockdown/knockout), chemical or enzyme treatment, and differential RT conditions. Orthogonal locus-specific detection methods are also necessary to validate the modification sites and status. In addition, synthetic modified spike-in controls preserve stoichiometry information during sequencing^122,183^. In addition, synthetic RNAs can also be developed for reducing false positives in sequencing-based technologies. For instance, an elegant study, which utilizes an unmodified IVT RNA library resembling the endogenous transcriptome as a negative control, can precisely and quantitatively map m^6^A and m^5^C through calibration^184^.

Nanopore direct RNA sequencing has been applied to the detection of several RNA modifications, showing promise for the simultaneous identification of distinct modifications in a single molecule. However, the accuracy and sensitivity of nanopore direct RNA modification sequencing are limited, and the simultaneous detection of different modifications is still unachieved. Further efforts should be made to improve the instrument to reduce the signal noise and develop robust computational analysis to identify multiple modifications simultaneously. Finally, the development of RNA modification detection technologies for low-input and single-cell samples is still an urgent need for facilitating understanding of the roles of RNA modifications in physiological and pathological processes. Improvement in integrating existing RNA modification detection strategies with single-cell RNA sequencing platforms shows promise. Collectively, recent progress in detection technologies promotes functional studies of RNA modifications, and we anticipate that further advancement will lead to a more comprehensive understanding of the epitranscriptome.

## References

[1] Boccaletto, P. et al. MODOMICS: a database of RNA modification pathways. 2021 update. *Nucleic Acids Res.* **50**, D231–D235 (2022). 10.1093/nar/gkab1083 PMC8728126 34893873

[2] Wang Y, Jia G (2020). Detection methods of epitranscriptomic mark N6-methyladenosine. Essays Biochem..

[3] Wang Y, Zhang X, Liu H, Zhou X (2021). Chemical methods and advanced sequencing technologies for deciphering mRNA modifications. Chem. Soc. Rev..

[4] Wiener D, Schwartz S (2021). The epitranscriptome beyond m(6)A. Nat. Rev. Genet..

[5] Owens MC, Zhang C, Liu KF (2021). Recent technical advances in the study of nucleic acid modifications. Mol. Cell.

[6] Helm M, Motorin Y (2017). Detecting RNA modifications in the epitranscriptome: predict and validate. Nat. Rev. Genet..

[7] Li X, Xiong X, Yi C (2016). Epitranscriptome sequencing technologies: decoding RNA modifications. Nat. Methods.

[8] Yang Y, Hsu PJ, Chen YS, Yang YG (2018). Dynamic transcriptomic m(6)A decoration: writers, erasers, readers and functions in RNA metabolism. Cell Res.

[9] Shi H, Wei J, He C (2019). Where, when, and how: context-dependent functions of RNA methylation writers, readers, and erasers. Mol. Cell.

[10] Barbieri I, Kouzarides T (2020). Role of RNA modifications in cancer. Nat. Rev. Cancer.

[11] Delaunay S, Frye M (2019). RNA modifications regulating cell fate in cancer. Nat. Cell Biol..

[12] Chen YS, Yang WL, Zhao YL, Yang YG (2021). Dynamic transcriptomic m(5) C and its regulatory role in RNA processing. Wiley Interdiscip. Rev. RNA.

[13] Shanmugam R (2015). Cytosine methylation of tRNA-Asp by DNMT2 has a role in translation of proteins containing poly-Asp sequences. Cell Disco..

[14] Shen H (2021). TET-mediated 5-methylcytosine oxidation in tRNA promotes translation. J. Biol. Chem..

[15] He C (2021). TET2 chemically modifies tRNAs and regulates tRNA fragment levels. Nat. Struct. Mol. Biol..

[16] Yang X (2017). 5-methylcytosine promotes mRNA export - NSUN2 as the methyltransferase and ALYREF as an m(5)C reader. Cell Res.

[17] Chen X (2019). 5-methylcytosine promotes pathogenesis of bladder cancer through stabilizing mRNAs. Nat. Cell Biol..

[18] Yang Y (2019). RNA 5-methylcytosine facilitates the maternal-to-zygotic transition by preventing maternal mRNA decay. Mol. Cell.

[19] Blanco S (2014). Aberrant methylation of tRNAs links cellular stress to neuro-developmental disorders. EMBO J..

[20] Gerber AP, Keller W (1999). An adenosine deaminase that generates inosine at the wobble position of tRNAs. Science.

[21] Seeburg PH, Hartner J (2003). Regulation of ion channel/neurotransmitter receptor function by RNA editing. Curr. Opin. Neurobiol..

[22] Wulff BE, Sakurai M, Nishikura K (2011). Elucidating the inosinome: global approaches to adenosine-to-inosine RNA editing. Nat. Rev. Genet..

[23] Arnez JG, Steitz TA (1994). Crystal structure of unmodified tRNA(Gln) complexed with glutaminyl-tRNA synthetase and ATP suggests a possible role for pseudo-uridines in stabilization of RNA structure. Biochemistry.

[24] Karijolich J, Yi C, Yu YT (2015). Transcriptome-wide dynamics of RNA pseudouridylation. Nat. Rev. Mol. Cell Biol..

[25] Newby MI, Greenbaum NL (2002). Investigation of overhauser effects between pseudouridine and water protons in RNA helices. Proc. Natl Acad. Sci. USA.

[26] Davis DR (1995). Stabilization of RNA stacking by pseudouridine. Nucleic Acids Res..

[27] King TH, Liu B, McCully RR, Fournier MJ (2003). Ribosome structure and activity are altered in cells lacking snoRNPs that form pseudouridines in the peptidyl transferase center. Mol. Cell.

[28] Liang XH, Liu Q, Fournier MJ (2007). rRNA modifications in an intersubunit bridge of the ribosome strongly affect both ribosome biogenesis and activity. Mol. Cell.

[29] Decatur WA, Fournier MJ (2002). rRNA modifications and ribosome function. Trends Biochem. Sci..

[30] Baudin-Baillieu A (2009). Nucleotide modifications in three functionally important regions of the Saccharomyces cerevisiae ribosome affect translation accuracy. Nucleic Acids Res..

[31] Jack K (2011). rRNA pseudouridylation defects affect ribosomal ligand binding and translational fidelity from yeast to human cells. Mol. Cell.

[32] Li X, Ma S, Yi C (2016). Pseudouridine: the fifth RNA nucleotide with renewed interests. Curr. Opin. Chem. Biol..

[33] Kariko K (2008). Incorporation of pseudouridine into mRNA yields superior nonimmunogenic vector with increased translational capacity and biological stability. Mol. Ther..

[34] Eyler DE (2019). Pseudouridinylation of mRNA coding sequences alters translation. Proc. Natl Acad. Sci. USA.

[35] Karijolich J, Yu YT (2011). Converting nonsense codons into sense codons by targeted pseudouridylation. Nature.

[36] Schevitz RW (1979). Crystal structure of a eukaryotic initiator tRNA. Nature.

[37] Helm M (1998). The presence of modified nucleotides is required for cloverleaf folding of a human mitochondrial tRNA. Nucleic Acids Res..

[38] Voigts-Hoffmann F (2007). A methyl group controls conformational equilibrium in human mitochondrial tRNA(Lys). J. Am. Chem. Soc..

[39] Dominissini D (2016). The dynamic N(1)-methyladenosine methylome in eukaryotic messenger RNA. Nature.

[40] Ballesta JP, Cundliffe E (1991). Site-specific methylation of 16S rRNA caused by pct, a pactamycin resistance determinant from the producing organism, Streptomyces pactum. J. Bacteriol..

[41] Waku T (2016). NML-mediated rRNA base methylation links ribosomal subunit formation to cell proliferation in a p53-dependent manner. J. Cell Sci..

[42] Li X (2017). Base-Resolution Mapping Reveals Distinct m(1)A Methylome in Nuclear- and Mitochondrial-Encoded Transcripts. Mol. Cell.

[43] Saikia M, Fu Y, Pavon-Eternod M, He C, Pan T (2010). Genome-wide analysis of N1-methyl-adenosine modification in human tRNAs. RNA.

[44] Chan CT (2010). A quantitative systems approach reveals dynamic control of tRNA modifications during cellular stress. PLoS Genet.

[45] Helm M, Alfonzo JD (2014). Posttranscriptional RNA Modifications: playing metabolic games in a cell’s chemical Legoland. Chem. Biol..

[46] Peifer C (2013). Yeast Rrp8p, a novel methyltransferase responsible for m1A 645 base modification of 25S rRNA. Nucleic Acids Res..

[47] Xiong X, Li X, Yi C (2018). N(1)-methyladenosine methylome in messenger RNA and non-coding RNA. Curr. Opin. Chem. Biol..

[48] Elliott BA (2019). Modification of messenger RNA by 2’-O-methylation regulates gene expression in vivo. Nat. Commun..

[49] Choi J (2018). 2’-O-methylation in mRNA disrupts tRNA decoding during translation elongation. Nat. Struct. Mol. Biol..

[50] Hoernes TP (2016). Nucleotide modifications within bacterial messenger RNAs regulate their translation and are able to rewire the genetic code. Nucleic Acids Res..

[51] Hoernes TP (2019). Eukaryotic Translation Elongation is Modulated by Single Natural Nucleotide Derivatives in the Coding Sequences of mRNAs. Genes (Basel).

[52] Ayadi L, Galvanin A, Pichot F, Marchand V, Motorin Y (2019). RNA ribose methylation (2’-O-methylation): Occurrence, biosynthesis and biological functions. Biochim. Biophys. Acta Gene Regul. Mech..

[53] Pandolfini L (2019). METTL1 Promotes let-7 MicroRNA Processing via m7G Methylation. Mol. Cell.

[54] Zhang LS (2019). Transcriptome-wide Mapping of Internal N(7)-Methylguanosine Methylome in Mammalian mRNA. Mol. Cell.

[55] Arango D (2018). Acetylation of Cytidine in mRNA Promotes Translation Efficiency. Cell.

[56] Kumbhar BV, Kamble AD, Sonawane KD (2013). Conformational preferences of modified nucleoside N(4)-acetylcytidine, ac4C occur at “wobble” 34th position in the anticodon loop of tRNA. Cell Biochem. Biophys..

[57] Ito S (2014). A single acetylation of 18 S rRNA is essential for biogenesis of the small ribosomal subunit in Saccharomyces cerevisiae. J. Biol. Chem..

[58] Jin G, Xu M, Zou M, Duan S (2020). The processing, gene regulation, biological functions, and clinical relevance of N4-acetylcytidine on RNA: a systematic review. Mol. Ther. Nucleic Acids.

[59] Dominissini D, Rechavi G (2018). N(4)-acetylation of Cytidine in mRNA by NAT10 Regulates Stability and Translation. Cell.

[60] Kellner S, Burhenne J, Helm M (2010). Detection of RNA modifications. RNA Biol..

[61] Stanley J, Vassilenko S (1978). A different approach to RNA sequencing. Nature.

[62] Gupta RC, Randerath K (1979). Rapid print-readout technique for sequencing of RNA’s containing modified nucleotides. Nucleic Acids Res.

[63] Keith G (1995). Mobilities of modified ribonucleotides on two-dimensional cellulose thin-layer chromatography. Biochimie.

[64] Zhao X, Yu YT (2004). Detection and quantitation of RNA base modifications. RNA.

[65] Grosjean H, Droogmans L, Roovers M, Keith G (2007). Detection of enzymatic activity of transfer RNA modification enzymes using radiolabeled tRNA substrates. Methods Enzymol..

[66] Grosjean H, Keith G, Droogmans L (2004). Detection and quantification of modified nucleotides in RNA using thin-layer chromatography. Methods Mol. Biol..

[67] Jia GF (2012). N6-Methyladenosine in nuclear RNA is a major substrate of the obesity-associated FTO (vol 7, pg 885, 2011). Nat. Chem. Biol..

[68] Nagarajan A, Janostiak R, Wajapeyee N (2019). Dot Blot Analysis for Measuring Global N(6)-Methyladenosine Modification of RNA. Methods Mol. Biol..

[69] Nees G, Kaufmann A, Bauer S (2014). Detection of RNA modifications by HPLC analysis and competitive ELISA. Methods Mol. Biol..

[70] Yoluc Y (2021). Instrumental analysis of RNA modifications. Crit. Rev. Biochem. Mol. Biol..

[71] Gaston KW, Limbach PA (2014). The identification and characterization of non-coding and coding RNAs and their modified nucleosides by mass spectrometry. RNA Biol..

[72] Kellner S (2014). Absolute and relative quantification of RNA modifications via biosynthetic isotopomers. Nucleic Acids Res.

[73] Thuring K, Schmid K, Keller P, Helm M (2016). Analysis of RNA modifications by liquid chromatography-tandem mass spectrometry. Methods.

[74] Bar-Yaacov D (2016). Mitochondrial 16S rRNA Is Methylated by tRNA Methyltransferase TRMT61B in All Vertebrates. PLoS Biol..

[75] Sharma S (2018). A single N(1)-methyladenosine on the large ribosomal subunit rRNA impacts locally its structure and the translation of key metabolic enzymes. Sci. Rep..

[76] Woo HH, Chambers SK (2019). Human ALKBH3-induced m(1)A demethylation increases the CSF-1 mRNA stability in breast and ovarian cancer cells. Biochim. Biophys. Acta Gene Regul. Mech..

[77] Jackman JE, Montange RK, Malik HS, Phizicky EM (2003). Identification of the yeast gene encoding the tRNA m1G methyltransferase responsible for modification at position 9. RNA.

[78] Lee C, Kramer G, Graham DE, Appling DR (2007). Yeast mitochondrial initiator tRNA is methylated at guanosine 37 by the Trm5-encoded tRNA (guanine-N1-)-methyltransferase. J. Biol. Chem..

[79] Bakin A, Ofengand J (1993). Four newly located pseudouridylate residues in Escherichia coli 23S ribosomal RNA are all at the peptidyltransferase center: analysis by the application of a new sequencing technique. Biochemistry.

[80] Datta B, Weiner AM (1991). Genetic evidence for base pairing between U2 and U6 snRNA in mammalian mRNA splicing. Nature.

[81] Herschlag D (2009). Biophysical, chemical, and functional probes of RNA structure, interactions and folding: Part A. Preface. Methods Enzymol..

[82] Maden BE, Corbett ME, Heeney PA, Pugh K, Ajuh PM (1995). Classical and novel approaches to the detection and localization of the numerous modified nucleotides in eukaryotic ribosomal RNA. Biochimie.

[83] Kiss T (2002). Small nucleolar RNAs: an abundant group of noncoding RNAs with diverse cellular functions. Cell.

[84] Yu YT, Shu MD, Steitz JA (1997). A new method for detecting sites of 2’-O-methylation in RNA molecules. RNA.

[85] Liu N (2013). Probing N6-methyladenosine RNA modification status at single nucleotide resolution in mRNA and long noncoding RNA. RNA.

[86] Li X (2015). Chemical pulldown reveals dynamic pseudouridylation of the mammalian transcriptome. Nat. Chem. Biol..

[87] Dong ZW (2012). RTL-P: a sensitive approach for detecting sites of 2’-O-methylation in RNA molecules. Nucleic Acids Res..

[88] Aschenbrenner J, Marx A (2016). Direct and site-specific quantification of RNA 2’-O-methylation by PCR with an engineered DNA polymerase. Nucleic Acids Res..

[89] Harcourt EM, Ehrenschwender T, Batista PJ, Chang HY, Kool ET (2013). Identification of a selective polymerase enables detection of N(6)-methyladenosine in RNA. J. Am. Chem. Soc..

[90] Castellanos-Rubio A (2019). A novel RT-QPCR-based assay for the relative quantification of residue specific m6A RNA methylation. Sci. Rep..

[91] Wang S (2016). N(6)-Methyladenine hinders RNA- and DNA-directed DNA synthesis: application in human rRNA methylation analysis of clinical specimens. Chem. Sci..

[92] Xiao Y (2018). An elongation- and ligation-based qPCR amplification method for the radiolabeling-free detection of locus-specific N(6) -methyladenosine modification. Angew. Chem. Int. Ed. Engl..

[93] Wang Y (2021). LEAD-m(6) A-seq for Locus-Specific Detection of N(6) -Methyladenosine and Quantification of Differential Methylation. Angew. Chem. Int. Ed. Engl..

[94] Qu HL, Michot B, Bachellerie JP (1983). Improved methods for structure probing in large RNAs: a rapid ‘heterologous’ sequencing approach is coupled to the direct mapping of nuclease accessible sites. Application to the 5’ terminal domain of eukaryotic 28S rRNA. Nucleic Acids Res..

[95] Lei Z, Yi C (2017). A Radiolabeling-Free, qPCR-Based Method for Locus-Specific Pseudouridine Detection. Angew. Chem. Int. Ed. Engl..

[96] Schurch S (2016). Characterization of nucleic acids by tandem mass spectrometry - The second decade (2004-2013): From DNA to RNA and modified sequences. Mass Spectrom. Rev..

[97] Jora M, Lobue PA, Ross RL, Williams B, Addepalli B (2019). Detection of ribonucleoside modifications by liquid chromatography coupled with mass spectrometry. Biochim. Biophys. Acta Gene Regul. Mech..

[98] Yuan BF (2017). Liquid chromatography-mass spectrometry for analysis of RNA adenosine methylation. Methods Mol. Biol..

[99] Suzuki T, Ikeuchi Y, Noma A, Suzuki T, Sakaguchi Y (2007). Mass spectrometric identification and characterization of RNA-modifying enzymes. Methods Enzymol..

[100] Akichika S, Suzuki T, Suzuki T (2021). Mass spectrometric analysis of mRNA 5’ terminal modifications. Methods Enzymol..

[101] Kurata T (2021). RelA-SpoT Homolog toxins pyrophosphorylate the CCA end of tRNA to inhibit protein synthesis. Mol. Cell.

[102] Bangs JD, Crain PF, Hashizume T, McCloskey JA, Boothroyd JC (1992). Mass spectrometry of mRNA cap 4 from trypanosomatids reveals two novel nucleosides. J. Biol. Chem..

[103] Guymon R, Pomerantz SC, Crain PF, McCloskey JA (2006). Influence of phylogeny on posttranscriptional modification of rRNA in thermophilic prokaryotes: the complete modification map of 16S rRNA of Thermus thermophilus. Biochemistry.

[104] Guymon R, Pomerantz SC, Ison JN, Crain PF, McCloskey JA (2007). Post-transcriptional modifications in the small subunit ribosomal RNA from Thermotoga maritima, including presence of a novel modified cytidine. RNA.

[105] Puri P (2014). Systematic identification of tRNAome and its dynamics in Lactococcus lactis. Mol. Microbiol..

[106] Suzuki T, Suzuki T (2014). A complete landscape of post-transcriptional modifications in mammalian mitochondrial tRNAs. Nucleic Acids Res..

[107] Mandal D (2010). Agmatidine, a modified cytidine in the anticodon of archaeal tRNA(Ile), base pairs with adenosine but not with guanosine. Proc. Natl Acad. Sci. USA.

[108] Miyauchi K, Kimura S, Suzuki T (2013). A cyclic form of N6-threonylcarbamoyladenosine as a widely distributed tRNA hypermodification. Nat. Chem. Biol..

[109] Kang BI (2017). Identification of 2-methylthio cyclic N6-threonylcarbamoyladenosine (ms2ct6A) as a novel RNA modification at position 37 of tRNAs. Nucleic Acids Res..

[110] Akichika S (2019). Cap-specific terminal N (6)-methylation of RNA by an RNA polymerase II-associated methyltransferase. Science.

[111] Ju YS (2011). Extensive genomic and transcriptional diversity identified through massively parallel DNA and RNA sequencing of eighteen Korean individuals. Nat. Genet..

[112] Bahn JH (2012). Accurate identification of A-to-I RNA editing in human by transcriptome sequencing. Genome Res..

[113] Ramaswami G (2012). Accurate identification of human Alu and non-Alu RNA editing sites. Nat. Methods.

[114] Peng Z (2012). Comprehensive analysis of RNA-Seq data reveals extensive RNA editing in a human transcriptome. Nat. Biotechnol..

[115] Ryvkin P (2013). HAMR: high-throughput annotation of modified ribonucleotides. RNA.

[116] Hauenschild R (2015). The reverse transcription signature of N-1-methyladenosine in RNA-Seq is sequence dependent. Nucleic Acids Res..

[117] Cozen AE (2015). ARM-seq: AlkB-facilitated RNA methylation sequencing reveals a complex landscape of modified tRNA fragments. Nat. Methods.

[118] Zheng G (2015). Efficient and quantitative high-throughput tRNA sequencing. Nat. Methods.

[119] Clark WC, Evans ME, Dominissini D, Zheng G, Pan T (2016). tRNA base methylation identification and quantification via high-throughput sequencing. RNA.

[120] Dai Q, Zheng G, Schwartz MH, Clark WC, Pan T (2017). Selective Enzymatic Demethylation of N(2),N(2) -Dimethylguanosine in RNA and Its Application in High-Throughput tRNA Sequencing. Angew. Chem. Int. Ed. Engl..

[121] Werner S (2020). Machine learning of reverse transcription signatures of variegated polymerases allows mapping and discrimination of methylated purines in limited transcriptomes. Nucleic Acids Res..

[122] Zhou H (2019). Evolution of a reverse transcriptase to map N(1)-methyladenosine in human messenger RNA. Nat. Methods.

[123] Incarnato D (2017). High-throughput single-base resolution mapping of RNA 2-O-methylated residues. Nucleic Acids Res..

[124] Bartoli, K. M., Schaening, C., Carlile, T. M. & Gilbert, W. V. Conserved Methyltransferase Spb1 Targets mRNAs for Regulated Modification with 2′-O-Methyl Ribose. Preprint at https://www.biorxiv.org/content/10.1101/271916v2 (2018).

[125] Aschenbrenner J (2018). Engineering of a DNA Polymerase for Direct m(6) A Sequencing. Angew. Chem. Int. Ed. Engl..

[126] Hong T (2018). Precise Antibody-Independent m6A Identification via 4SedTTP-Involved and FTO-Assisted Strategy at Single-Nucleotide Resolution. J. Am. Chem. Soc..

[127] Wang Y, Xiao Y, Dong S, Yu Q, Jia G (2020). Antibody-free enzyme-assisted chemical approach for detection of N(6)-methyladenosine. Nat. Chem. Biol..

[128] Sakurai M (2014). A biochemical landscape of A-to-I RNA editing in the human brain transcriptome. Genome Res..

[129] Squires JE (2012). Widespread occurrence of 5-methylcytosine in human coding and non-coding RNA. Nucleic Acids Res..

[130] Edelheit S, Schwartz S, Mumbach MR, Wurtzel O, Sorek R (2013). Transcriptome-wide mapping of 5-methylcytidine RNA modifications in bacteria, archaea, and yeast reveals m5C within archaeal mRNAs. PLoS Genet.

[131] Khoddami V (2019). Transcriptome-wide profiling of multiple RNA modifications simultaneously at single-base resolution. Proc. Natl Acad. Sci..

[132] Huang T, Chen W, Liu J, Gu N, Zhang R (2019). Genome-wide identification of mRNA 5-methylcytosine in mammals. Nat. Struct. Mol. Biol..

[133] Legrand C (2017). Statistically robust methylation calling for whole-transcriptome bisulfite sequencing reveals distinct methylation patterns for mouse RNAs. Genome Res..

[134] Carlile TM (2014). Pseudouridine profiling reveals regulated mRNA pseudouridylation in yeast and human cells. Nature.

[135] Lovejoy AF, Riordan DP, Brown PO (2014). Transcriptome-wide mapping of pseudouridines: pseudouridine synthases modify specific mRNAs in S. cerevisiae. PLoS One.

[136] Schwartz S (2014). Transcriptome-wide mapping reveals widespread dynamic-regulated pseudouridylation of ncRNA and mRNA. Cell.

[137] Enroth C (2019). Detection of internal N7-methylguanosine (m7G) RNA modifications by mutational profiling sequencing. Nucleic Acids Res..

[138] Finet O (2021). Transcription-wide mapping of dihydrouridine reveals that mRNA dihydrouridylation is required for meiotic chromosome segregation. Mol. Cell.

[139] Sas-Chen A (2020). Dynamic RNA acetylation revealed by quantitative cross-evolutionary mapping. Nature.

[140] Shu X (2020). A metabolic labeling method detects m(6)A transcriptome-wide at single base resolution. Nat. Chem. Biol..

[141] Hu, L. et al. m6A RNA modifications are measured at single-base resolution across the mammalian transcriptome. *Nat. Biotechnol.* 1–10 (2022). 10.1038/s41587-022-01243-z PMC9378555 35288668

[142] Birkedal U (2015). Profiling of ribose methylations in RNA by high-throughput sequencing. Angew. Chem. Int. Ed. Engl..

[143] Marchand V, Blanloeil-Oillo F, Helm M, Motorin Y (2016). Illumina-based RiboMethSeq approach for mapping of 2’-O-Me residues in RNA. Nucleic Acids Res..

[144] Krogh N (2016). Profiling of 2’-O-Me in human rRNA reveals a subset of fractionally modified positions and provides evidence for ribosome heterogeneity. Nucleic Acids Res..

[145] Zhu Y, Pirnie SP, Carmichael GG (2017). High-throughput and site-specific identification of 2’-O-methylation sites using ribose oxidation sequencing (RibOxi-seq). RNA.

[146] Dai Q (2017). Nm-seq maps 2’-O-methylation sites in human mRNA with base precision. Nat. Methods.

[147] Marchand V (2018). AlkAniline-Seq: profiling of m(7) G and m(3) C RNA modifications at single nucleotide resolution. Angew. Chem. Int. Ed. Engl..

[148] Lin S (2018). Mettl1/Wdr4-mediated m(7)G tRNA methylome is required for normal mRNA translation and embryonic stem cell self-renewal and differentiation. Mol. Cell.

[149] Cui J, Liu Q, Sendinc E, Shi Y, Gregory RI (2021). Nucleotide resolution profiling of m3C RNA modification by HAC-seq. Nucleic Acids Res..

[150] Marchand V (2020). HydraPsiSeq: a method for systematic and quantitative mapping of pseudouridines in RNA. Nucleic Acids Res..

[151] Dominissini D (2012). Topology of the human and mouse m6A RNA methylomes revealed by m6A-seq. Nature.

[152] Meyer KD (2012). Comprehensive analysis of mRNA methylation reveals enrichment in 3’ UTRs and near stop codons. Cell.

[153] Li X (2016). Transcriptome-wide mapping reveals reversible and dynamic N(1)-methyladenosine methylome. Nat. Chem. Biol..

[154] Delatte B (2016). RNA biochemistry. Transcriptome-wide distribution and function of RNA hydroxymethylcytosine. Science.

[155] Linder B (2015). Single-nucleotide-resolution mapping of m6A and m6Am throughout the transcriptome. Nat. Methods.

[156] Chen K (2015). High-resolution N(6) -methyladenosine (m(6) A) map using photo-crosslinking-assisted m(6) A sequencing. Angew. Chem. Int. Ed. Engl..

[157] Malbec L (2019). Dynamic methylome of internal mRNA N(7)-methylguanosine and its regulatory role in translation. Cell Res..

[158] Safra M (2017). The m1A landscape on cytosolic and mitochondrial mRNA at single-base resolution. Nature.

[159] Sun H (2021). m(6)Am-seq reveals the dynamic m(6)Am methylation in the human transcriptome. Nat. Commun..

[160] Hussain S (2013). NSun2-mediated cytosine-5 methylation of vault noncoding RNA determines its processing into regulatory small RNAs. Cell Rep..

[161] Khoddami V, Cairns BR (2013). Identification of direct targets and modified bases of RNA cytosine methyltransferases. Nat. Biotechnol..

[162] Meyer KD (2019). DART-seq: an antibody-free method for global m(6)A detection. Nat. Methods.

[163] Tegowski M, Flamand MN, Meyer K (2022). D. scDART-seq reveals distinct m(6)A signatures and mRNA methylation heterogeneity in single cells. Mol. Cell.

[164] Knutson SD, Arthur RA, Johnston HR, Heemstra JM (2020). Selective enrichment of A-to-I edited transcripts from cellular RNA using endonuclease V. J. Am. Chem. Soc..

[165] Imanishi M, Tsuji S, Suda A, Futaki S (2017). Detection of N(6)-methyladenosine based on the methyl-sensitivity of MazF RNA endonuclease. Chem. Commun. (Camb.).

[166] Garcia-Campos MA (2019). Deciphering the “m(6)A code” via antibody-independent quantitative profiling. Cell.

[167] Zhang Z (2019). Single-base mapping of m(6)A by an antibody-independent method. Sci. Adv..

[168] Wang Y, Zhao Y, Bollas A, Wang Y, Au KF (2021). Nanopore sequencing technology, bioinformatics and applications. Nat. Biotechnol..

[169] Begik O (2021). Quantitative profiling of pseudouridylation dynamics in native RNAs with nanopore sequencing. Nat. Biotechnol..

[170] Furlan M (2021). Computational methods for RNA modification detection from nanopore direct RNA sequencing data. RNA Biol..

[171] Wan YK, Hendra C, Pratanwanich PN, Goke J (2021). Beyond sequencing: machine learning algorithms extract biology hidden in Nanopore signal data. Trends Genet.

[172] Jenjaroenpun P (2021). Decoding the epitranscriptional landscape from native RNA sequences. Nucleic Acids Res..

[173] Leger A (2021). RNA modifications detection by comparative Nanopore direct RNA sequencing. Nat. Commun..

[174] Parker MT (2020). Nanopore direct RNA sequencing maps the complexity of Arabidopsis mRNA processing and m(6)A modification. Elife.

[175] Liu H (2019). Accurate detection of m(6)A RNA modifications in native RNA sequences. Nat. Commun..

[176] Smith AM, Jain M, Mulroney L, Garalde DR, Akeson M (2019). Reading canonical and modified nucleobases in 16S ribosomal RNA using nanopore native RNA sequencing. PLoS One.

[177] Price AM (2020). Direct RNA sequencing reveals m(6)A modifications on adenovirus RNA are necessary for efficient splicing. Nat. Commun..

[178] Garalde DR (2018). Highly parallel direct RNA sequencing on an array of nanopores. Nat. Methods.

[179] Gao Y (2021). Quantitative profiling of N(6)-methyladenosine at single-base resolution in stem-differentiating xylem of Populus trichocarpa using nanopore direct RNA sequencing. Genome Biol..

[180] Pratanwanich PN (2021). Identification of differential RNA modifications from nanopore direct RNA sequencing with xPore. Nat. Biotechnol..

[181] Kim D (2020). The architecture of SARS-CoV-2 transcriptome. Cell.

[182] Miladi, M. et al. The landscape of SARS-CoV-2 RNA modifications. Preprint at https://www.biorxiv.org/content/10.1101/2020.07.18.204362v1 (2020).

[183] Koh CWQ, Goh YT, Goh WSS (2019). Atlas of quantitative single-base-resolution N(6)-methyl-adenine methylomes. Nat. Commun..

[184] Zhang Z (2021). Systematic calibration of epitranscriptomic maps using a synthetic modification-free RNA library. Nat. Methods.

